# Regional expiratory time constants in severe respiratory failure estimated by electrical impedance tomography: a feasibility study

**DOI:** 10.1186/s13054-018-2137-3

**Published:** 2018-09-21

**Authors:** Christian Karagiannidis, Andreas D. Waldmann, Péter L. Róka, Tina Schreiber, Stephan Strassmann, Wolfram Windisch, Stephan H. Böhm

**Affiliations:** 1 Department of Pneumology and Critical Care Medicine, Cologne-Merheim Hospital, Kliniken der Stadt Köln gGmbH, Witten/Herdecke University Hospital, Ostmerheimer Strasse 200, D-51109 Cologne, Germany; 2 Swisstom AG, Schulstrasse 1, 7302 Landquart, Switzerland; 3 Budapest University of Technology and Economics, Budapest, Hungary; 4 Klinik und Poliklinik für Anästhesiologie und Intensivtherapie, Universitätsmedizin Rostock, Schillingallee 35, D-18057 Rostock, Germany

**Keywords:** Severe COPD, Exacerbation, ARDS, Expiratory time constant, Flow limitation, Electrical impedance tomography

## Abstract

**Background:**

Electrical impedance tomography (EIT) has been used to guide mechanical ventilation in ICU patients with lung collapse. Its use in patients with obstructive pulmonary diseases has been rare since obstructions could not be monitored on a regional level at the bedside. The current study therefore determines breath-by-breath regional expiratory time constants in intubated patients with chronic obstructive pulmonary disease (COPD) and acute respiratory distress syndrome (ARDS).

**Methods:**

Expiratory time constants calculated from the global impedance EIT signal were compared to the pneumatic volume signals measured with an electronic pneumotachograph. EIT-derived expiratory time constants were additionally determined on a regional and pixelwise level. However, regional EIT signals on a single pixel level could in principle not be compared with similar pneumatic changes since these measurements cannot be obtained in patients. For this study, EIT measurements were conducted in 14 intubated patients (mean Simplified Acute Physiology Score II (SAPS II) 35 ± 10, mean time on invasive mechanical ventilation 36 ± 26 days) under four different positive end-expiratory pressure (PEEP) levels ranging from 10 to 17 cmH_2_O. Only patients with moderate-severe ARDS or COPD exacerbation were included into the study, preferentally within the first days following intubation.

**Results:**

Spearman’s correlation coefficient for comparison between EIT-derived time constants and those from flow/volume curves was between 0.78 for tau (τ) calculated from the global impedance signal up to 0.83 for the mean of all pixelwise calculated regional impedance changes over the entire PEEP range. Furthermore, Bland-Altman analysis revealed a corresponding bias of 0.02 and 0.14 s within the limits of agreement ranging from − 0.50 to 0.65 s for the aforementioned calculation methods. In addition, exemplarily in patients with moderate-severe ARDS or COPD exacerbation, different PEEP levels were shown to have an influence on the distribution pattern of regional time constants.

**Conclusions:**

EIT-based determination of breath-by-breath regional expiratory time constants is technically feasible, reliable and valid in invasively ventilated patients with severe respiratory failure and provides a promising tool to individually adjust mechanical ventilation in response to the patterns of regional airflow obstruction.

**Trial registration:**

German Trial Register DRKS 00011650, registered 01/31/17.

**Electronic supplementary material:**

The online version of this article (10.1186/s13054-018-2137-3) contains supplementary material, which is available to authorized users.

## Background

Ventilatory support in patients with severe respiratory failure is steadily increasing in modern intensive care medicine. Although substantial progress has been made, mortality in ICU patients still remains high [1, 2]. Individualizing treatment to specific patient needs is suggested to improve the outcome particularly in ICU patients on ventilatory support [3]. One important limitation, however, towards individualized ventilatory support is the lack of technology to monitor regional lung function continuously at the bedside. While lung function has been monitored by the analysis of flow and volume curve for decades, this reflects global lung function only [4, 5]. Accordingly, these global pneumatic signals cannot distinguish differences in regional pathophysiology. In this regard, ventilator settings may have distinct or even opposite effects on different lung regions due to oscillating air/Pendelluft [6, 7], atelectasis or overdistension, which typically remains undetected in daily clinical practice.

Modern diagnostic techniques such as transpulmonary pressure measurement [8] or electrical impedance tomography (EIT) [9–15] when used to guide mechanical ventilation have been shown to improve gas exchange in patients with ARDS [16–20]. In particular, EIT is a unique technique to continuously determine regional differences in atelectasis and overdistension [21–27]. However, until now airflow limitation, which is a hallmark of obstructive pulmonary diseases such as asthma, chronic obstructive pulmonary disease (COPD) or bronchiolitis has never been monitored breathwise on a regional level. The estimation of regional expiratory time constants by EIT has the potential to provide a new dimension for bedside monitoring of patients with obstructive lung diseases or even for small airway alterations in ARDS.

Although the expiratory time constants measured at the tube at airway opening summarizes time constants from different regions of the lung, it provides clinically useful information on the mechanical properties of the entire respiratory system [28, 29]. Different approaches to calculating the global expiratory time constant have been suggested in the past. Marini et al. [30] proposed a method dividing the total expiratory volume by the peak expiratory flow. Brunner et al. [31] modified this approach for cases with incomplete exhalation and could precisely determine the expiratory time constants for passive exhalation by adding a correction factor for incomplete exhalation. Although regional compliance (C) and resistance (R) can intra-individually vary considerably between different lung regions, the aforementioned approaches are capable of measuring a single compartment model only. To overcome this limitation and to estimate the heterogeneity of passive lung deflation, Henderson et al. [32] divided the global flow-volume curve into a sequence of five consecutive volume segments and calculated the expiratory time constant for each segment separately. All these techniques reflect the global expiratory time constants to which all lung areas contribute. In regard to regional lung function measurement, Vogt et al. [33] assessed the spatial and temporal heterogeneity of ventilation by EIT during pulmonary function testing in differentially aged subjects without lung disease and in patients with COPD [34]. Their results indicate EIT to be capable of providing information on the spatial and temporal distribution of the emptying characteristics during a single forced exhalation. Nevertheless, the method is thus not suited for the continuous monitoring of mechanically ventilated patients.

To date, regional online airflow obstruction measurements in patients with the most severe lung failure have yet to be determined [35, 36]. Therefore, the aim of the present study was to develop, evaluate and validate an EIT-based method to measure regional expiratory time constants on a breath by breath basis and pixelwise level. Fourteen intubated and invasively ventilated patients with severe respiratory failure resulting from moderate to severe ARDS, COPD exacerbation or lung transplant rejection were studied, and time constants were analyzed offline afterwards. Although regional changes in ventilation are the primary aim of the method, the global pneumatic vs. EIT-derived signals had to be validated, since pneumatic changes on a comparable pixel level could not be obtained at all using current technology.

## Methods

### Patients and ethics

The study was approved by the Ethics Committee of the Witten/Herdecke University and registered at the German Clinical Trial Register and the World Health Organization (WHO) trial register (DRKS00011650/ U1111–1192-0396). Patients with COPD, leading to exacerbation with invasive mechanical ventilation and those with pneumonia and/or moderate to severe ARDS ventilated at different PEEP levels were enrolled in this study. Diagnosis of COPD was made by two independent pulmonologists in accordance to the medical history, flow characteristics of the mechanical ventilation, x-ray of the chest and known pre-diagnosis from pulmonologists. During the study protocol PEEP was titrated by decrements from 16/17 to 14/15 to 12/13 and 10/11 cmH_2_O applying a tidal volume of 6 ml/kg predicted body weight (PBW). Every step lasted 30 min. Patients were deeply sedated (Richmond Agitation Sedation Scale (RASS) score − 4) without any spontaneous breathing effort. Pneumatic volume measurements and the corresponding EIT signal were obtained at the end of each 30-min period for post-hoc breath by breath analysis. The study was conducted in the early phase of the disease as soon as written informed consent could be obtained from the legal representative.

### Pneumatic measurements by electronic pneumotachograph

Acting as reference, the global flow signal was measured with a sampling rate of 1 kHz at the distal end of the tracheal tube using the hot-wire anemometry V-Meter (EKU, Leiningen, Germany) and integrated to obtain volumes.

### EIT measurements

**Fig. 1:**
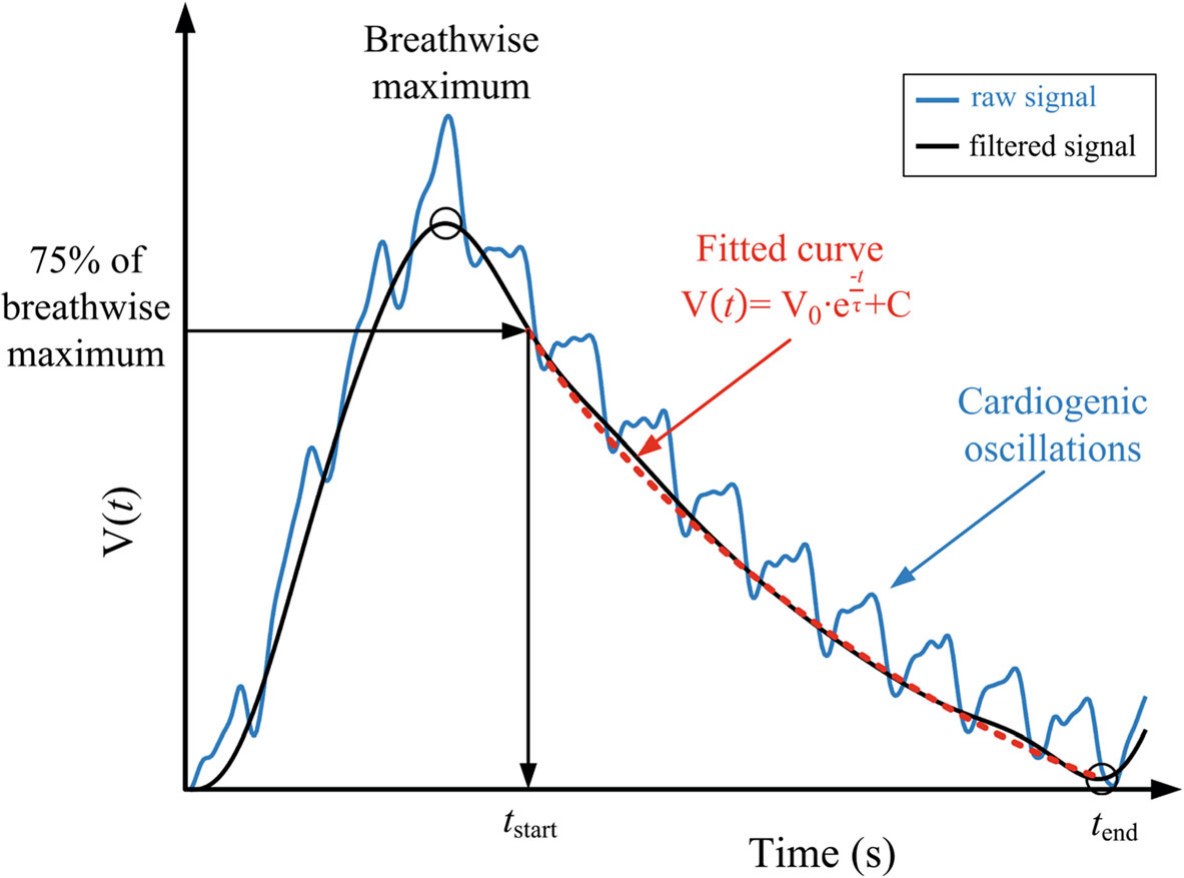
Algorithm for calculating regional time constant *τ*_reg_. Of note, curve fitting starts at the time when 75% of peak signal is reached (see “Methods”). V(*t*): volume at time point *t,* V_0_ volume at start of expiration, *t* the time from the start at end-inspiration to the end of expiration, *τ* the expiratory time constant and C the end expiratory volume

**Fig. 2:**
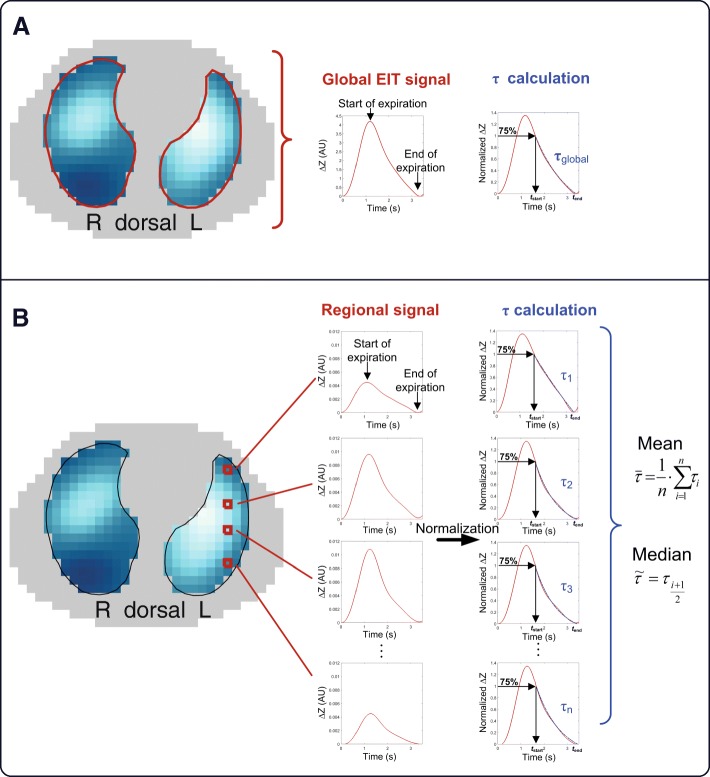
Tau calculation from global (**a**) vs. mean or median (**b**) of regional tau. ΔZ, impedance change. EIT, electrical impedance tomography

EIT data were measured using the Swisstom BB^2^ (Swisstom AG, Landquart, Switzerland). Each patient’s thorax circumference was measured, and the textile electrode belt of appropriate size fastened along the 6th intercostal space [37]. Fifty cross-sectional impedance images were recorded every second for a period of 10 min under stable conditions. In addition, lung and thorax contours derived from patient-specific computer tomography (CT) scans, which were performed routinely during the course of the disease, were projected into the EIT images (Additional file 1: Figure S2). CT images were analyzed in the inspiration phase. Accordingly, only signals from the CT-defined lung regions were analyzed (Figs. 1 and 2) [38]. EIT images were low-pass filtered to suppress cardiac-related impedance changes. The cutoff frequency for the low-pass filter was adjusted for each patient individually according to the patient’s heart rate. Time constants were calculated by exponential fitting for every single pixel. If the single pixel had no exponential fit, e.g. pixel from the heart or atelectasis without ventilation, this pixel was excluded from further analysis. Furthermore, the global impedance (ΔZ(*t*)global) signal was calculated as the sum of all signals at any point of time. The start and end of expiration were determined, respectively from the global EIT signal. Then within this time sequence, for each single pixel the 75% amplitude was calculated as the start of the curve fitting (Fig. 2b) and a local minimum was used to define the end of expiration. However, the global signal is only used to define the time sequence of the expiration but start and end was defined pixelwise.

## Calculation of regional time constant τ_reg_

Passive expiration resembles an exponential decay:

$$ V(t)={V}_0\bullet {e}^{\frac{-t}{\tau }}+C $$

where V(*t*) is the volume at that time point *t,* V_0_ is the volume at the start of expiration, *t* the time from the start at end-inspiration to the end of expiration, *τ* the expiratory time constant and C the end-expiratory volume (Fig. 1). The time constant *τ* represents the time for V(*t*) to exhale 2/3 of its volume, with 3*τ* defining the time to reach at least 95% of complete exhalation. In mechanically ventilated patients, especially in COPD, exhalation may be incomplete during a tidal breath and, thus, in this case expiratory flow does not reach zero before the start of the next breath. The exhalation of each lung region correlates with an exponential decay in volume, which can be calculated. However, the expiratory volume curve is not typically exponential in mechanically ventilated patients [39] since the onset of expiration is mostly dominated by inertial effects and differs from the following parts of the exhalation curve. To overcome this problem, Lourens et al. [28] advised starting the analysis at 75% of the breath-wise maximum and therefore skip the first part of the exhalation curve for calculation. Furthermore, they demonstrated that expiratory time constants determined from the expiratory flow-volume curve using 75% of tidal volume were in closest agreement with the time constant obtained with interrupter measurements. Subsequently, the first 25% of the local signal amplitude was omitted; thus, τ was calculated from the remaining 75% of the pneumatic signal by fitting an exponential curve to the pneumatic signal and the global EIT signal, and to the signal of each pixel within the lung regions (Fig. 2). EIT signals stemming from lung areas with low or no ventilation, or non-exponential signals were excluded from *τ* calculations.

**Fig. 3:**
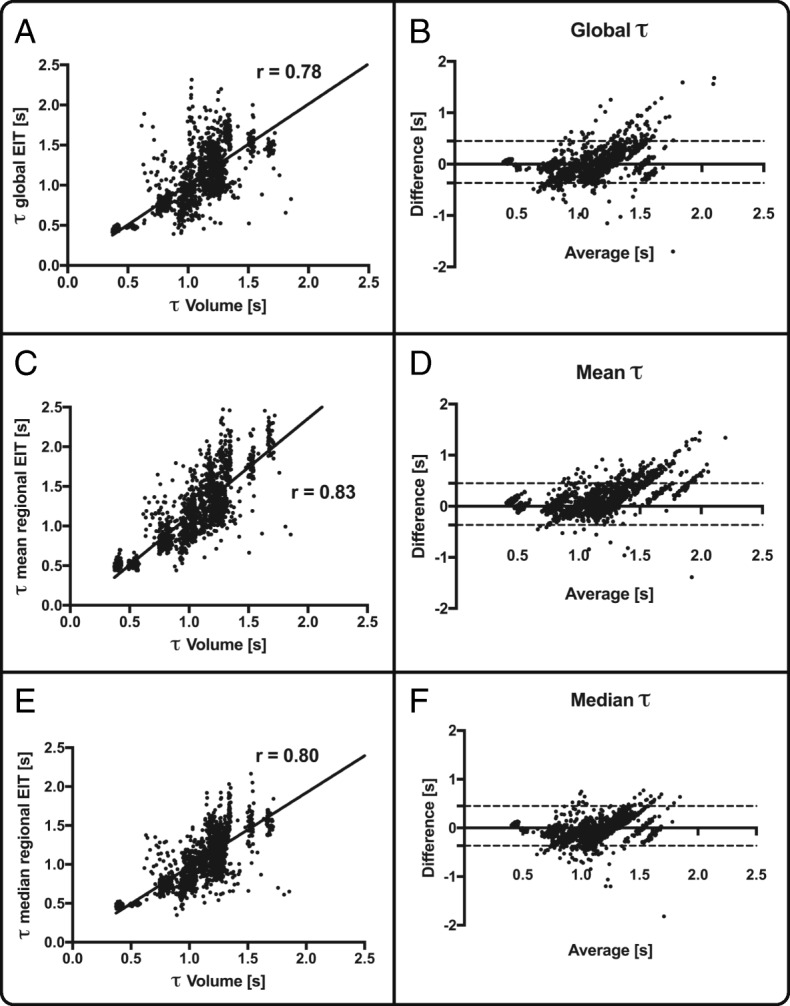
Spearman correlation *r* between global tau calculation and volume signal (**a**), mean regional electrical impedance tomography (EIT)-derived tau (**c**) and median regional EIT-derived tau (**e**). The corresponding Bland-Altman analysis is displayed in **b**, **d** and **f**

The expiratory time constants were assessed at airway opening in the whole respiratory system by curve fitting of the volume curve and compared with the time constants calculated from impedance changes over time measured by EIT (Figs. 1 and 2). Validation was performed by comparing the global expiratory time constant gained from the volume signal with the global signal from EIT, since no regional pneumatic information can be determined on a comparable pixel level. Figure 3 demonstrates the correlation between the pneumatic signal and all EIT-derived signals for every single breath recorded during the timeframe and for all PEEP levels. To estimate the robustness of our method, we calculated the regional coefficient of variation (CV) for all breaths as standard deviation divided by the mean (Additional file 2: Figure S1).

### Statistics

Correlation was tested between the time constants derived from the pneumatic volume curve and EIT-derived time constants by calculating Spearman’s correlation coefficient. Every single breath was compared using both methods. Bland-Altman analysis was performed on the same data using GraphPad Prism version 7 (GraphPad Software, La Jolla, CA, USA).

## Results

Fourteen patients with severe respiratory failure with a mean SAPS II score of 35 ± 10 and a mean time on invasive mechanical ventilation of 36 ± 26 days were included in the study. All but one patient suffered from pneumonia as the leading cause of respiratory failure from different underlying diseases (Table 1).

**Table 1:** baseline characteristics of the patients

Age (years)	57 ± 15
Gender (female/male)	4/10
Pneumonia	13/14
History of COPD (GOLD II-IV)^a^	7/14
ARDS^a^	8/14
SAPS II at admission	35 ± 10
LTOT before	4/14
Home NIV before	2/14
Active smoking	11/14
Mortality	3/14
Days on mechanical ventilation	36 ± 26
Days in ICU	41 ± 37
Respiratory system mechanics and blood gas analysis	
pH day 1	7.24 ± 0.1
PaCO_2_ day 1 (mmHg)	72 ± 20
PaO_2_ day 1 (mmHg)	82 ± 19
P/F ratio day 1 (mmHg)	124 ± 41
PEEP day 1 (cmH_2_O)	14 ± 2
P_insp_ day 1 (cmH_2_O)	30 ± 4
Tidal volume day 1 (ml)	470 ± 88
PEEP day 7 (cmH_2_O)	13 ± 2
P_insp_ day 7 (cmH_2_O)	26 ± 4
Tidal volume day 7 (ml)	450 ± 101

Baseline characteristics of all patients included in the study

*COPD* chronic obstructive pulmonary disease, *GOLD* Global Initiative for Chronic Obstructive Lung Disease, *LTOT* long-term oxygen therapy, *SAPS* Simplified Acute Physiology Score, *NIV* non-invasive ventilation, *PaCO*_*2*_ partial arterial pressure of carbon dioxide, *PaO*_*2*_ partial arterial pressure of oxygen, *PEEP* positive end-expiratory pressure, *P*_*insp*_ peak inspiratory pressure

^a^May occur at the same time

**Fig. 4:**
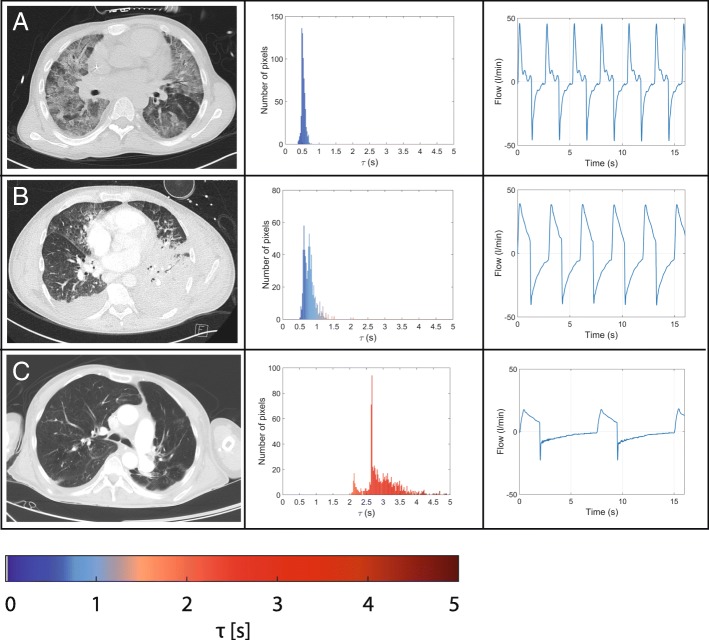
Typical examples of frequency distribution of regional *τ* values calculated in a stiff lung (**a**), in acute respiratory distress syndrome with pneumonia (**b**) and in chronic obstructive pulmonary disease (**c**). From left to right: computed tomography scan, histogram of *τ* regional values and flow curve

**Fig. 5:**
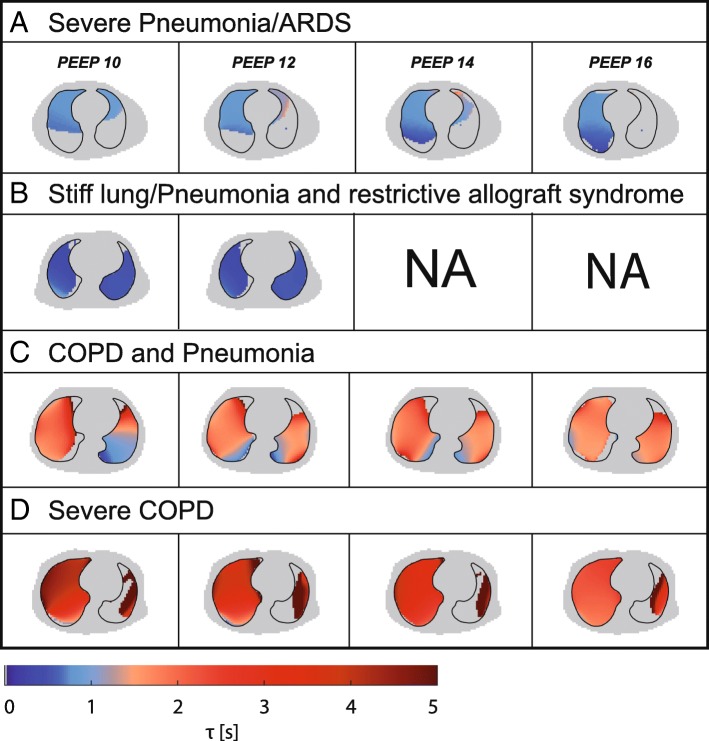
Typical examples of τ determined at different positive end-expiratory pressure (PEEP) levels in pneumonia/acute respiratory distress syndrome (ARDS) (**a**), in stiff lungs (**b**) and in chronic obstructive pulmonary disease (COPD) (**c** and **d**) with its regional distribution. NA = not applicable

**Fig. 6:**
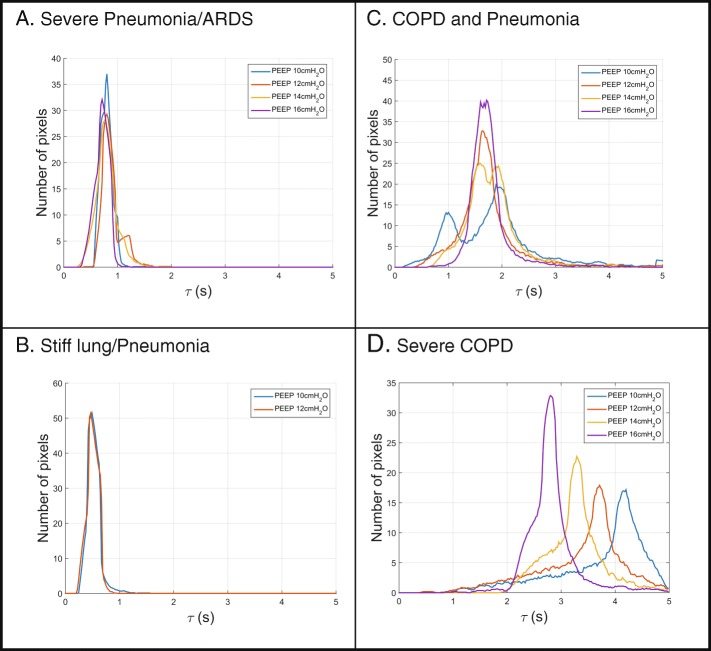
Typical examples of τ determined at different positive end-expiratory pressure (PEEP) levels in pneumonia/acute respiratory distress syndrome (ARDS) (**a**), in stiff lungs (**b**) and in chronic obstructive pulmonary disease (COPD) (**c** and **d**) displayed as pixel-wise histograms

The EIT-derived expiratory time constant was calculated in different lung diseases and conditions of invasive mechanical ventilation (Figs. 4, 5 and 6). Exemplarily, Fig. 4 shows three main diseases, i.e. a stiff lung, ARDS and COPD, demonstrating a huge variation in expiratory time constants and spatial distribution. Comparison of calculation methods (time constants derived from global EIT signal vs. mean or median of regional expiratory time constants) revealed strong correlation (Fig. 3) in all cases with correlation coefficients ranging from 0.72 (PEEP10/11) to 0.81 (PEEP 14/15; Fig. 3 and Additional file 3: Table S1). The overall correlation for the entire PEEP range was 0.83 (*p* < 0.001). The lowest bias and limits of agreement according to the Bland-Altman analysis were for the global EIT signal and the median of the regional expiratory time constants. In detail, overall the correlation was 0.78 with a bias of 0.02 s within the limits of agreement ranging from − 0.50 to 0.53 s for *τ* calculated from the global impedance signal. Correlation for the *τ* calculated as the mean of all pixelwise regional impedance changes was 0.83 with a higher bias of 0.14 s within the limits ranging from − 0.37 to 0.65 s. Furthermore, correlation for the median *τ* derived from the same regional impedance changes was 0.80 (bias − 0.03, limits of agreement − 0.50 to 0.22 s).

Typical patient examples are given in Figs. 4 and 5, showing expiratory time constants lasting longer than 2 s in patients with severe COPD (Fig. 4c) and very short ones in restrictive lung diseases such as in restrictive allograft syndrome with pneumonia (Fig. 4a). Histograms revealed a narrow homogeneous frequency distribution of *τ* values in patients with restrictive lung diseases such as restrictive allograft syndrome or ARDS and a widespread inhomogeneous distribution with *τ* values ranging from 2 to 5 s in patients with COPD (Fig. 4c).

## Discussion

This is the first study to demonstrate that breath-by-breath measurements of regional expiratory time constants by EIT are technically feasible, reliable and valid in invasively ventilated patients with severe respiratory failure. In particular, EIT as used in the current study has provided new insights into the spatial and temporal distribution of airflow limitation also exemplarily shown in response to different PEEP settings in ARDS and COPD (Figs. 5 and 6). Thereby, the assessment of regional expiratory time constants clearly added previously unavailable information to the results achieved by the standard analysis derived from global flow and volume curves.

The algorithm to calculate the EIT-derived expiratory time constant was validated in patients with severe respiratory failure and was highly correlated (correlation coefficient approximately 0.80) with the pneumatic volume signal, demonstrating the usability of the EIT-derived expiratory time constants. EIT measurements were reproducible and robust (Additional file 2: Figure S1) under changing ventilation strategies, demonstrating the clinical usability of the system. Nevertheless, limits of agreement alternate depending on the type of disease, airflow obstruction, resulting time constant and at certain PEEP levels (Additional file 3: Table S1), in part reaching 0.5 s. However, in the author’s opinion, first the trend over time is more important than absolute numbers in daily clinical practice; second, in a real-time breath-by-breath analysis, single outliers as shown in the Bland-Altmann analysis, have a minor impact, especially for long time constants, since most of the values are in an accurate range. This reflects the advantage of EIT-based real-time measurements at the bedside with every breath. Third, the accuracy of the measurement can be further improved simply by averaging 5–10 breaths over time without losing the regional information.

Our study shows the physiological influence of different PEEP levels on regional airflow limitations. These data are in line with current animal experiments, demonstrating that whole lung measures of strain do not accurately represent regional pulmonary mechanics [40]. Furthermore, Pulletz et al. demonstrated different time constants measured by EIT in patients with ARDS, comparable to the example given in Fig. 4 [41], which in principle also holds true for preterm infants [42]. Thereby, beneficial and potentially harmful effects of PEEP were demonstrated in both ARDS and COPD alike. The illustrated example curves even demonstrate contradictory effects within a single patient, highlighting the importance of regional analysis. Furthermore, EIT effectively demonstrated the inhomogeneous emptying characteristics of different lung regions in severe COPD compared to more homogenous lungs in some patients with ARDS. Therefore, EIT-based expiratory time constant analysis could serve as a clinically useful bedside adjunct for defining the optimal PEEP level and ventilation strategy in different lung conditions. Furthermore, the breath-wise analysis opens the opportunity to determine regional trends over time to estimate the best overall ventilator settings.

Concerning the absolute values, the described method estimates the potential time the lung would need to fully deflate. Therefore, the time constant appears to be longer than clinically predicted but reflects the behavior of small airways and the difficulty of their respective dependent lung units to become fully deflated. One might speculate that the trending of *τ* over time might be as valuable as the absolute *τ* values per se. Furthermore, all patient examples given in the manuscript reflect images of regional lung function, which were matched with the morphological alterations in CT scan and global flow analysis, thereby, underlining the reliability of the method for use in clinical practice.

The study has, however, some limitations. First, the chosen algorithm to calculate the expiratory time constant is based on the hypothetical assumption of a complete expiration, which may not be achieved clinically at least in some patients with severe airflow obstruction. Therefore, in this scenario expiratory time constants as calculated by EIT might be higher than the actual time a lung region needs for expiration, an effect that becomes more pronounced with longer time constants and higher breathing frequencies. Second, for validation purposes the current study evaluates the expiratory time constant in deeply sedated patients without any breathing efforts, but not in spontaneously breathing patients. However, even in spontaneously breathing patients with high breathing frequency, EIT may gather results comparable to standard lung function testing where breathing commands are given. Third, in general, EIT with the current technology does not cover the time constants from all regions of the lung. However, in the current study at least 10 cm from up to down are covered with the EIT belt [9]. Finally, repetitive measurements in the same patient to obtain even more robust data were not ethically possible due to the severity of the disease.

## Conclusions

In conclusion, EIT-based breath-by-breath determination of regional expiratory time constant is technically feasible, reliable and valid in invasively ventilated patients with severe respiratory failure. In addition, different PEEP levels were exemplarily shown to have an influence on the distribution pattern of regional time constants in different types of acute severe lung failure. Future studies should determine the clinical impact of measuring airflow obstruction on a regional basis in patients with acute or chronic respiratory failure requiring mechanical ventilation.

## Additional files

Additional file 1: **Figure S2.** Image reconstruction and segmentation of corresponding CT scan. (PDF 6665 kb)

Additional file 2: **Figure S1.** Coefficient of variation in EIT-derived *τ* calculations in 14 patients. (PDF 1276 kb)

Additional file 3: **Table S1.** Correlation, bias and limits of agreement for different PEEP levels. (DOCX 14 kb)

## Abbreviations

ARDS: Acute respiratory distress syndrome
COPD: Chronic obstructive pulmonary disease
CT: Computed tomography
EIT: Electrical impedance tomography
PEEP: Positive end-expiratory pressure
RASS: Richmond Agitation Sedation Scale
SAPS: Simplified Acute Physiology Score
