# Supplementary figures and images for: Regional expiratory time constants in severe respiratory failure estimated by electrical impedance tomography: a feasibility study
Source: Crit Care. 2018 Sep 21;22:221. doi: 10.1186/s13054-018-2137-3 (PMC6148957; doi:10.1186/s13054-018-2137-3)

Supplemental Figure 2

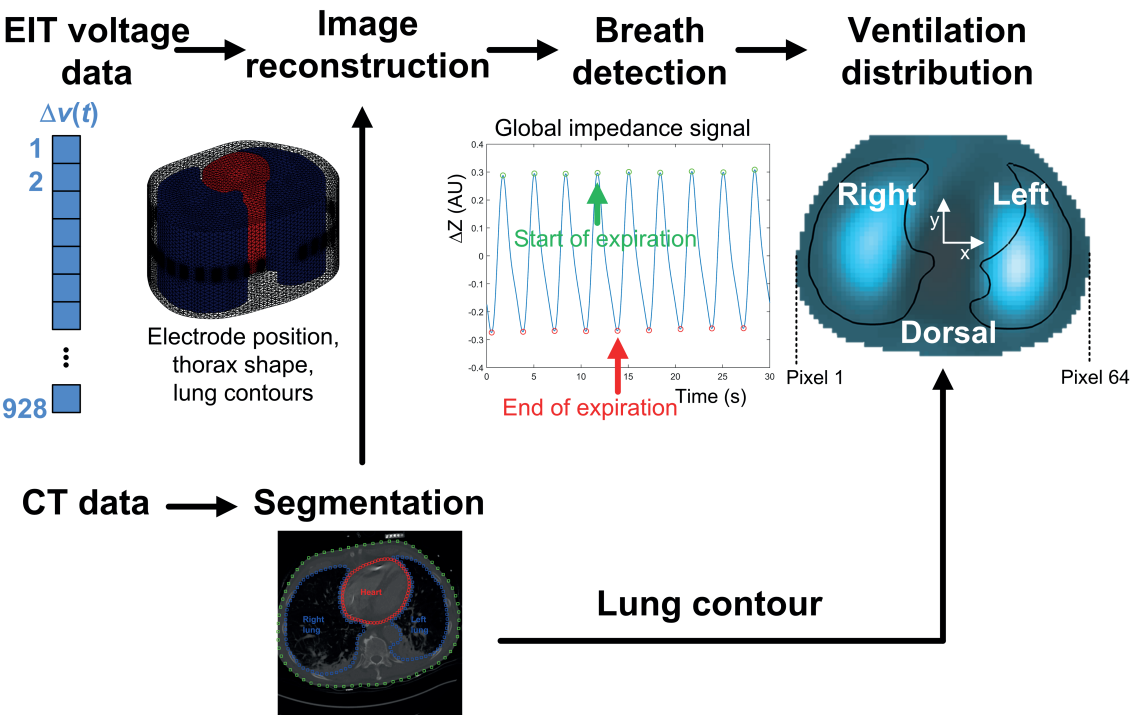

Supplement: Supplementary file 1 — Figure S2. Image reconstruction and segmentation of corresponding CT scan. (PDF 6665 kb) [file 13054_2018_2137_MOESM1_ESM.pdf]

Supplemental Figure 1

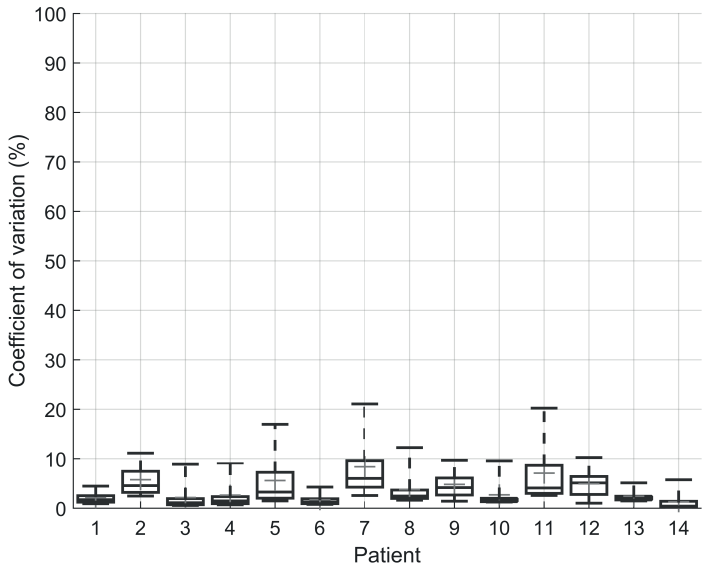

Supplement: Supplementary file 2 — Figure S1. Coefficient of variation in EIT-derived τ calculations in 14 patients. (PDF 1276 kb) [file 13054_2018_2137_MOESM2_ESM.pdf]
