# Supplementary material for: Regional expiratory time constants in severe respiratory failure estimated by electrical impedance tomography: a feasibility study
Source: Crit Care. 2018 Sep 21;22:221. doi: 10.1186/s13054-018-2137-3 (PMC6148957; doi:10.1186/s13054-018-2137-3)
Supplement: Supplementary file 3 — Table S1. Correlation, bias and limits of agreement for different PEEP levels. (DOCX 14 kb) [file 13054_2018_2137_MOESM3_ESM.docx]

| PEEP Level | Correlation Coefficient  Spearman r for single PEEP level EIT mean regional | Bias in corresponding Bland-Altmann Analysis | Limits of agreement in corresponding Bland-Altmann Analysis |
| --- | --- | --- | --- |
| 10/11 | 0.72 | 0.31 | -0.27 to 0.89 |
| 12/13 | 0.80 | 0.12 | -0.34 to 0.56 |
| 14/15 | 0.81 | 0.09 | -0.43 to 0.61 |
| 16/17 | 0.76 | 0.13 | -0.31 to 0.57 |
| *Overall* | |  |  |
| *10-17* | ***0.83*** | ***0.14*** | **-0.37 to 0.65** |

Supplemental Table 1: correlation, bias, limits of agreement for different PEEP level

Legend: Correlation, bias and limits of agreement for expiratory time constants calculated from EIT vs. volume measurements in regard to individual PEEP level.
